# A Risk and Hazard Analysis Model for the Production Process of a New Meat Product Blended With Germinated Green Buckwheat and Food Safety Awareness

**DOI:** 10.3389/fnut.2022.902760

**Published:** 2022-06-22

**Authors:** Zhibek Atambayeva, Almagul Nurgazezova, Maksim Rebezov, Galiya Kazhibayeva, Samat Kassymov, Diana Sviderskaya, Sandugash Toleubekova, Zhanna Assirzhanova, Rysqul Ashakayeva, Zukhra Apsalikova

**Affiliations:** ^1^Department of Technology of Food Production and Biotechnology, Shakarim University of Semey, Semey, Kazakhstan; ^2^V.M. Gorbatov Federal Research Center for Food Systems, Russian Academy of Sciences (RAS), Moscow, Russia; ^3^Department of Biotechnology, S. Toraighyrov Pavlodar State University, Pavlodar, Kazakhstan; ^4^Faculty of Foundation, Innovative University of Eurasia, Pavlodar, Kazakhstan

**Keywords:** HACCP, food safety, hazard analysis, ISO 22000:2018, risk assessment, public health, mixed meat, patties

## Abstract

This study was specifically designed for a small-scale meat processing enterprise “DARIYA” to set up a specific HACCP plan for the new product (patties) made from mixed horsemeat with vegetable components developed in the Department of Technology of Food Production and Biotechnology, Shakarim University of Semey. Critical control points (CCPs) were identified and applied in the HACCP plan. The different hazards were detected at each processing step, whereas each CCP in the HACCP plan was identified and accompanied with the appropriate significant hazard, critical limit, monitoring of the CCP, and corrective actions, confirming that the enterprise has fully employed the HACCP methodology and ISO 22000:2018. Our results indicate that during almost 1 year following the implementation of ISO 22000:2018, the coliform level of tested patties significantly dropped (*p* < 0.05) after 6 months of implementation (coliform count dropped from 4.4 MPN/g to 1.8 MPN/g). The rapid screening of the bacterial count, heavy metals, pesticide residue, and physical contamination levels also improved monitoring assertiveness, allowing them to deal with foreseeable issues linking to resources and guarantee product quality. Cesium-137 was recorded as 5.4 ± 2.9627 Bq/kg in horsemeat and 6.7 ± 2.7045 in poultry. The activity of cesium-137 did not exceed the MAC. This result discloses that prompt screening is the foremost and necessary step for small enterprises. According to this study, the “acceptance of raw materials” is the most important CCP, and their control, particularly in small-scale meat processing enterprises, can actually prevent many negative outcomes. The implementation of both standards improved food quality by declining the flaw rates for patties, and the number of flow inconsistencies needed for correction in the process also dropped significantly (*p* < 0.05), demonstrating that safety and quality points were improving. If the application of the HACCP plan were to continue over an extended period of time, the Food Safety Management System's (FSMS) benefits would be more substantial improvements to a greater number of items being monitored. The process of implementing HACCP principles and ISO 22000:2018 could be arduous but achievable enough to be used in small industries with significant outcomes.

## Introduction

The rapid changes in the conditions of existence of modern society and advances in science and technology over the past few decades have raised our standard of living and have accelerated the pace of human life. On this basis, a vast and thriving market for foods with “functional” or health benefits has emerged. More and more residents of megalopolises are inclined to buy ready-made culinary products in the supermarket or dine “outside the home” and are ready to increase their expenses for the saved time. The main criteria that determine the choice of a ready-made dish by the consumer are high quality, homemade taste, convenience, and functionality. The growing demand for semi-finished meat products and ready-made meals stimulates manufacturers to increase production volumes and expand the range of these products. The use of traditional raw materials in different combinations, the mixture of minced meat with raw materials of animal and vegetable origin, the introduction of food additives, the use of modern equipment, and advanced technologies allows not only diversifying the list of manufactured products, giving the product different flavoring shades, but also improving the technological properties of minced meat, increasing its biological value (1–4).

Horsemeat and its products can be categorized as health-promoting foods with high protein, iron, and omega-3 fatty acids, along with low-fat content. Thanks to this, horsemeat is rightfully considered a dietary meat. Experts say that the absorption of horsemeat in the body is about eight times faster than the absorption of beef (5, 6). According to nutritionists, the benefit of horsemeat is that the fats it contains are somewhere in between animal and vegetable fats. Due to its ability to lower blood cholesterol, horsemeat is an excellent product for regulating metabolic processes in the body, and its low-fat content makes it quite suitable for diets aimed at weight loss. Horsemeat is hypoallergenic, and in addition, it is rich in vitamins E and B, and due to this feature, the intake of horsemeat would improve blood circulation (7–9).

Adherents to a healthy lifestyle and proper nutrition are undoubtedly familiar with such a product as green buckwheat. Although a preponderance of the population is skeptical about it, associating the word “green” with immaturity. Green buckwheat is a unique type of cereal that has both nutritional value and a supply of nutrients. The principal value of this cereal is that it is an unprocessed product. As a result, it is also called live buckwheat, a gluten-free food with valuable phytochemicals that provide significant health benefits. Buckwheat holds high nutritional value due to bioactive compounds such as polysaccharides, proteins, amino acids, flavonoids, dietary fiber, vitamins, and minerals (10). In terms of health benefits, buckwheat and its by-products contain antioxidants capable of scavenging free radicals (11–13). The germinated seeds or sprouts are nutritionally better than their original seeds, with greater levels of nutrients, lower amounts of compounds that interfere with the absorption of nutrients, and increased protein and starch digestibility (14, 15). Therefore, scientists are always challenged with the mission of finding resources of irreplaceable food components through the use of various types of raw materials and their combination, allowing them to produce high-quality products with increased nutritional and biological values. Meat processing adds value to food and exhibits the final products with specific tastes, color, flavor, or texture, which diverse from fresh meat. Processed meat with different additives proposes diversity to the meat food sector, delivering the combined effect of nutritious food with exceptional taste. Providing people with high-quality and environmentally safe food products can be guaranteed by developing the production of food by creating a sustainable food future (16–19).

Food security is becoming progressively vital in food production, and the implementation of HACCP is essential for all small- and medium-sized enterprises. The foremost cause is the exposure of products to bacteriological, physical, and chemical hazards. The Codex Alimentarius Commission defines CCPs as a series of steps to avoid, lessen, or remove threats; they are incorporated with International Standard ISO 22000:2018 (20), which was published on June 19, 2018, “Management systems in the field of food safety. Requirements for organizations participating in the food chain.” It states that all companies must conduct a risk analysis to categorize substantial hazards. The ISO 22000:2018 entered into force in Kazakhstan on 1 December 2019. The ISO 22000:2018 certification is needed to ensure the development and effective functioning of the food safety management system aimed at the production of food safety for consumers, to demonstrate compliance with applicable food safety laws and regulations to all interested parties, and to assess and analyze customer requirements to increase customer satisfaction. Prerequisite Programs (PRPs) and Operational Prerequisite Programs (OPRPs) are tasks for enterprise employees and are implied in the HACCP program documentation. The fulfillment of these tasks ensures control at every stage of the technological process during the preparation of food products, and these programs aim to reduce the influence of hazardous factors on finished products. Since many food products today cross national borders on several occasions, international standards are essential to ensure the safety of the global supply chain. ISO 22000:2018 is a food safety management system standard that applies to any organization in the “field-to-fork” food supply chain, enabling the enterprise to produce high-quality, safe products while providing consumers with visual confirmation that the manufacturer is responsible for sanitary requirements and safety. For food manufacturing, the HACCP program is currently known as the highest approach to food security control (21–23). In food manufacturing, one of the 12 application steps for the HACCP methodology is hazard identification, and it is considered critical. It also agrees with the first principle of HACCP and ISO 22000:2018, which calls for the implementation of hazard analysis. HACCP systems aim to recognize, assess, and control hazards. The application of HACCP has allowed Kazakhstan enterprises to take part in international marketplaces and, thus, effectively expand their markets and profits.

The purpose of this study was to establish and examine the implementation of HACCP and ISO 22000:2018 by conducting a hazard analysis in a small-scale meat processing enterprise for the production of patties from mixed horsemeat with vegetable components developed in the Department of Technology of Food Production and Biotechnology, Shakarim University of Semey. Furthermore, to identify CCPs and establish a preventive measure system will lead to a safer production of meat and its products.

## Materials and Methods

### Study Subject and Description

This study was carried out at the small-scale meat products enterprise (SMPE – “Dariya”) in Semey city, East Kazakhstan. The enterprise produces meat products like sausages, patties, and cutlets. To meet the demands of its customers and partners, the company was planning to obtain the ISO 220000:2018 and HACCP certification for food safety. The key aim of the study was to establish and implement food safety management (ISO 22000:2018 and HACCP) in the enterprise for the production of patties from mixed horsemeat with vegetable components developed in the Department of Technology of Food Production and Biotechnology, Shakarim University of Semey. The products focus on the domestic market, with a view to entering the markets of neighboring countries after certification.

### Steps of the Implementation HACCP Plan

**Figure 1:**
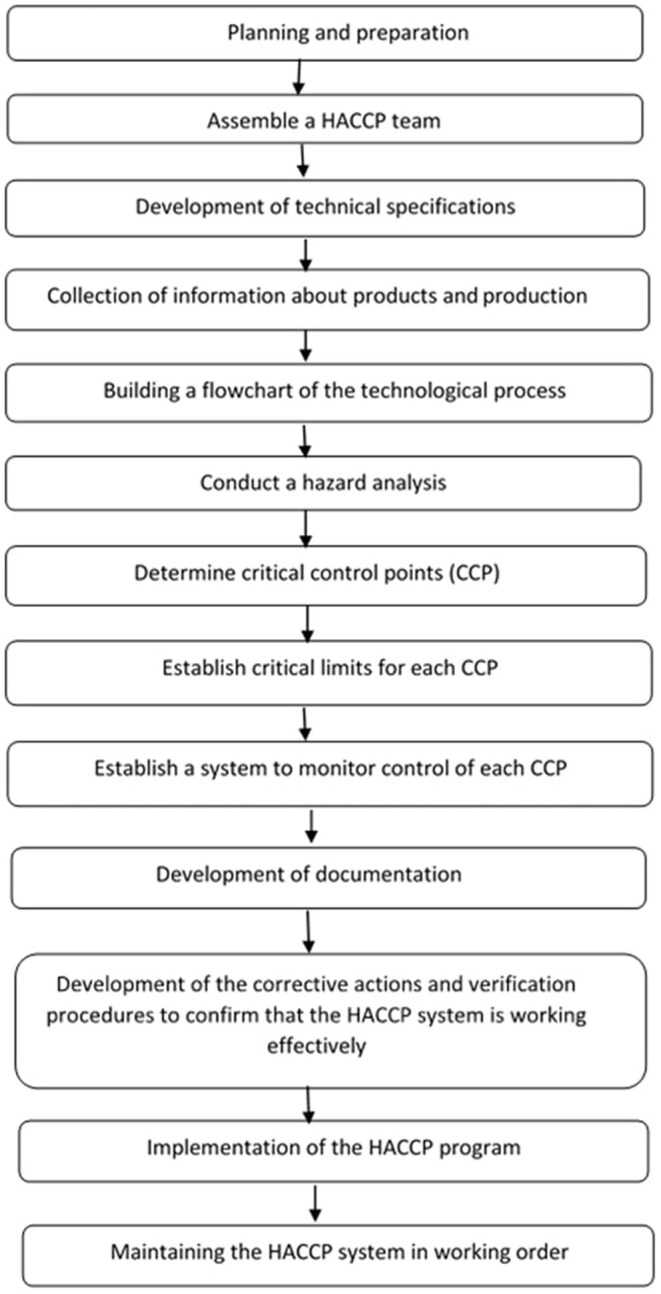
Algorithm for implementing the principles of HACCP.

In this study, the HACCP plan was completed according to Chapter 8 of ISO 2200:2018, and the seven basic principles of HACCP are employed by the Codex Alimentarius Commission and implemented in production lines. The algorithm of study in the development of a food safety system based on the standards of HACCP and ISO 22000:2018 for small- and medium-sized enterprises is shown in Figure 1. First, the Prerequisite Programs (PRP) that linked procedures and risk evaluation methods to identify significant hazards were presented, followed by applying a decision tree to differentiate between CCPs and operational prerequisite programs (OPRPs).

### Assembling a HACCP Team (Chapter 5.3 of ISO 22000:2018)

For the implementation and development of the HACCP system at the enterprise, it is compulsory to form a working group of employees with various specializations who have the proper knowledge about specific products, work experience, and methodology for developing an effective plan for the application of the HACCP system at the enterprise. The HACCP working group was multidisciplinary and composed of seven people, namely, a HACCP team head, a production technological engineer, a university attendant in charge of student practice, a sanitation unit engineer, a quality assurance unit manager, a hygiene manager, and a HACCP team secretary; all work was handled according to the requirements of the standard (20, 24). “DARIYA” enterprise is an organization where the practical assignments are performed with the food engineering students (as an internship in training programs and integrated educational programs). A university attendant in charge of student practice was included in the HACCP team as a part of an educational program. A working group was assembled to evaluate and manage all processes involved in implementing the system and applying FSMS through the help of food security consultants. A highly qualified team with responsibility can make it possible to increase the quality stages of all procedures and products. The enterprise's general manager had been actively supporting the insertion and modification of regulatory requirements in response to the newly introduced ISO 22000:2018, in combination with the incorporation of a HACCP system. However, the main incentives mentioned for implementing a certified system were the assured trust of consumers, the prevention of food risks, and the improvement of the company's image.

## Materials and Analysis

### Acquisition of Raw Materials and Analysis Items

Horsemeat (grade I), chicken (thighs), grain, vegetables, and spices were purchased from the local market and transported in the secured plastic bags. Upon arrival at the lab, they were washed, cleaned, and packed individually in polyethylene bags and kept at 4°C in a fridge before use. The study examined mixed meat patties with sprouted green buckwheat and compared the data obtained between May 2017 and November 2018 and between May 2018 and November 2019 (before and after the employment of ISO 22000:2018). Coliforms, *Escherichia coli*, and foodborne pathogens, including *Salmonella*, and *Listeria monocytogenes* (MPG/g) in both raw and final products were determined. The final products were tested for the TPC (total plate count, the quantity of mesophilic aerobic and facultative anaerobic microorganisms), heavy metals (arsenic, cadmium, mercury, and lead), radionuclide (cesium-137), antibiotics, and pesticide residue components. Heavy metals, radionuclides, antibiotics, and pesticide residues were examined by a third-party testing laboratory. All chemical and biological tests were carried out in accordance with official procedures published by the Standards of the Republic of Kazakhstan.

### Production of Patties

The preparation of patties was done in the mini meat patty production line, which was equipped with a meat grinder/chopper (JR-100D, China), and a single-paddled meat mixer (JBW-50, China). The prepared horsemeat (40%), chicken (thighs) (25%), horse fat (7%), sprouted green buckwheat (5%), cabbage (4%), and onion (3%) are first ground in a grinder with 2–3 mm diameter lattice holes. Then, the ground components are blended in the meat mixer with eggs (2%), sprouted green buckwheat flour (2.5%), black pepper (0.3%), salt (1.3%), and water (10%) until an evenly mixed texture is formed. The mixture for the patties is portioned into the desired weight (100 g each, 10 cm diameter/1.5 cm thickness), shaped using a molding device, placed on trays, and frozen at −29 to 30°C for 1 h.

### Packaging, Cooling, and Storing

After 1 h, the patties were packed in 500 g lots in vacuum plastic bags, labeled, and kept in a freezer at −18°C for required analyses (a minimum of 24 h).

### Microbiological Analysis

#### Quick Screening Method

For rapid analysis, we used 3M™ Petrifilm™ test plates. These plates are ready-made nutrient media for the microbiological analysis of raw materials, semi-finished products, finished products, and environmental objects in the food and beverage industry. For the TPC, the PetrifilmTM (RAC) was used. First, the top film of the plate was raised, and 1 ml of the taster was positioned on the center of the bottom film by means of a pipette perpendicular to the surface of the plate. Next, the top film is placed down on the sample carefully, avoiding any air bubbles formation. The liquid is evenly distributed within the circumference by the dispenser. Finally, the plate was stacked clear side up and incubated in a 37 ± 1°C incubator for 24 h. Following the completion of the procedure, the number of colonies on the test plates (red) was completed three times. For simultaneous detection of *E. coli* and coliform bacteria, the 3M™ Petrifilm™ (EC) test plates were used. The presence of a chromogenic substrate β-glucuronidase facilitates colony counting for the detection of *E. coli*. First, the top film of the plate was raised, and 1 ml of the sample was placed on the center of the bottom film using a pipette perpendicular to the surface of the plate. Next, the top film is placed down on the sample carefully, avoiding any air bubbles formation. The liquid is evenly distributed within the circumference by the dispenser. Finally, the plate was stacked clear side up and incubated in a 37 ± 1°C incubator for 24 h. After the procedure was completed, the number of colonies on test plates (blue – *E. coli* and red – coliforms) was counted thrice. For *Salmonella* detection, we used the 3M™ Petrifilm™ *Salmonella* Express System (3M™ Petrifilm™ SALX). First, 25 g of sample was added to 225 ml of *Salmonella* selective enrichment medium (3M *Salmonella* enrichment base and 3M *Salmonella* enrichment supplement). Incubated for 18 h at 41.5°C. Then, 0.1 ml of the culture liquid from the enrichment medium was transferred to 10 ml of Rappaport-Vassiliadis RV R10 selective broth and incubated for 8 h at 41.5°C. Next, loop onto the surface of the 3M™ Petrifilm™ *Salmonella* Express (SALX) plate and incubated for 24 ± 2 h at 41.5°C. Finally, the confirmation disk was placed on Petrifilm for biochemical confirmation of *Salmonella* and incubated for 4 h at 41.5°C and noted the results using the interpretation guide. For *Listeria monocytogenes* detection, 3M™ Petrifilm™ (EL) Environmental Test Plate was used. Procedures are the same as described in the detection of TPC. *Listeria* colonies are reddish-violet. Reading the results after 28 h was performed thrice.

### Study Approach and Effectiveness of Enterprise Risk Management

**Figure 2:**
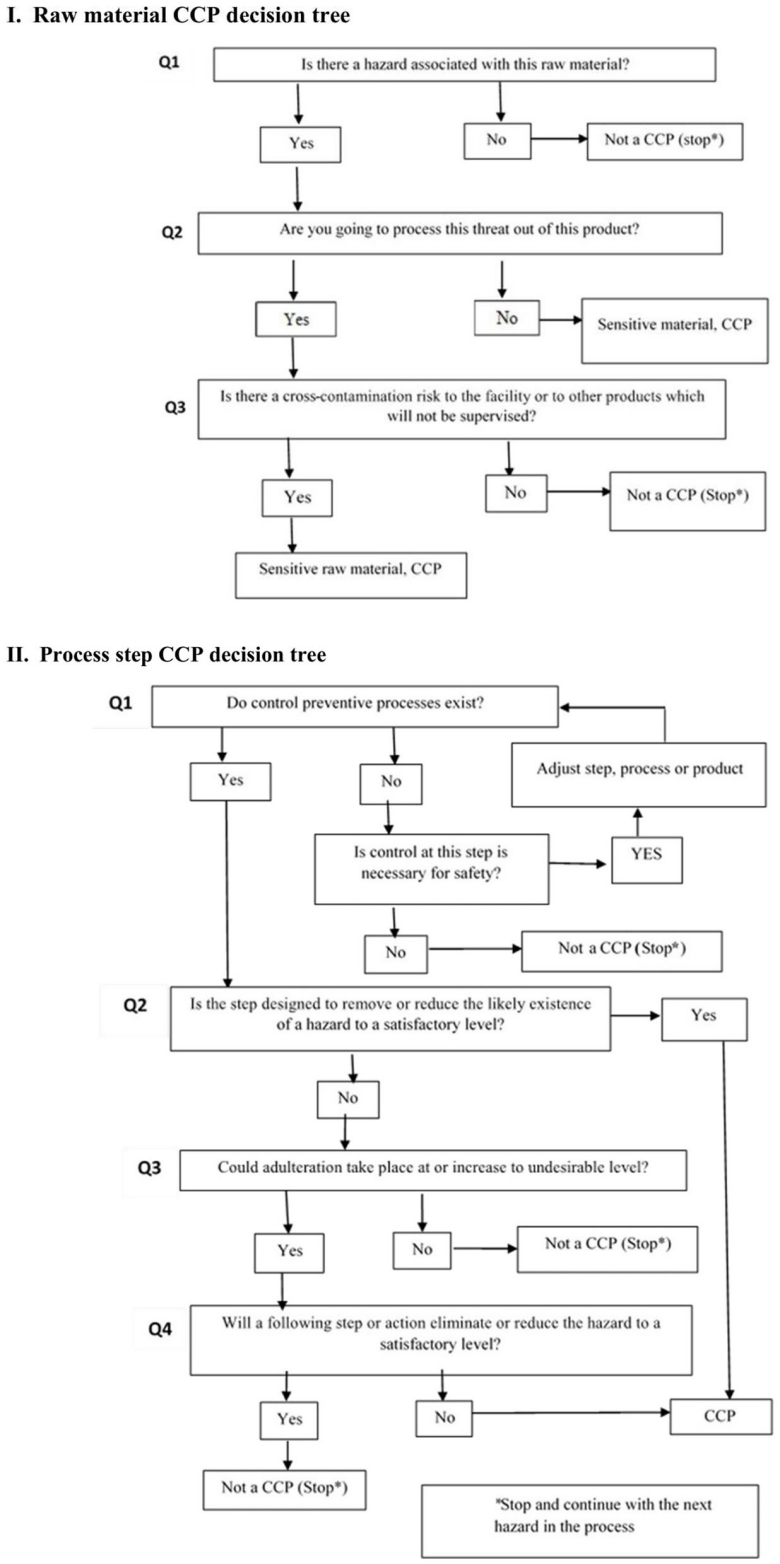
CCP process decision tree (I – Raw material CCP decision tree; II – Process step CCP decision tree).

The study was divided into the following four parts: the formation of the HACCP team, the evaluation of data (gathering and analysis of information on the incoming products), identifying main hazards, critical control point determination, and the HACCP program chart. However, the main steps of the development of the HACCP idea were founded on ISO 22000:2018 (17), and the principles of HACCP in the production of patties were applied, as given in Figure 1. The analysis was planned to identify the potential threats (biological, chemical, and physical) and the possibility of their occurrence throughout the production. The determination of CCPs involves identifying and characterizing the crucial hazards after answering the well-known questions of the decision tree to decide whether they fit CCP (Figure 2). The critical limit in each CCP is demonstrated according to the reference and standard; corrective action is instigated to improve or fix a CCP; verification processes are conducted to guarantee that the HACCP plan is working effectively to implement in the production line. Documentation must be recorded accurately during the whole procedure in HACCP. Due to the size of the functioning plant and financial concerns, the enterprise we worked with does not have in-house laboratories. Rapid testing of raw materials to ensure product safety and effectiveness may be inaccurate, posing a risk of infected product incidents. To recheck rapid test results, our samples of raw materials and final products were tested in the accredited microbiological laboratory of Semey city. The development of the HACCP scheme started after discussing the manufacturing process of patties. The application of the plan was intended to cover all steps of the production, starting from receiving raw materials and finishing with the end product. Therefore, to make stronger the enterprise monitoring effectiveness and to create periodic rotation planning and monitoring system records that take into account any food safety incidents, inconsistencies in the production line, and manufacturer supply stability, the enterprise needs a work plan that will set the pattern of successful work. The determination of the presence of heavy metals (lead, cadmium, mercury, and arsenic), radionuclide (cesium-137), antibiotics, and pesticide residues was done by another test laboratory. During study time, heavy metals were tested one at a time every 3 months to prevent the finished product from any chemical hazards (25–27). The requirements for the safety of food products and processes of their development, production, circulation, utilization, and disposal, as stipulated by the legislation of the Republic of Kazakhstan in the area of food safety, are essential for the producers of food products.

It is required to know all the properties of the components of the product to perform an accurate hazard analysis and develop a HACCP plan (Table 1). Different elements of the final product that might affect its safety need to be considered.

**Table 1:** The list of ingredients and their nutritional values in patties.

**Ingredients**	**Nutritional value (kcal/100 g)**
Horse meat	133
Chicken meat (thighs)	215
Sprouted buckwheat grain	343
Cabbage	24
Onion	35
Eggs	164
Flour from sprouted buckwheat grain	335
Black pepper	255
Salt	0
Water	0

### Statistical Analysis

Statistica 12.0 (STATIS-TICA, 2014; StatSoft Inc., Tulsa, OK, USA) was performed to determine the dissimilarities between samples and were calculated using the ANOVA method, followed by a Student's *t*-test to determine the significance of the differences between the two groups. Differences were interpreted to be statistically important at *p* ≤ 0.05.

## Results and Discussions

### Initial Steps in Applying the HACCP System

#### Description of the Product and Intended Uses (Chapters 8.5.1.2–8.5.1.4 of ISO 22000:2018)

The food safety team preceded a complete description of the product, categorizing its important safety information like composition, physical/chemical structure, microbicidal conduct, packaging, shelf-life, storage condition, method of delivery, and intended uses (28–31). The product description for patty “Shygys” is given in Table 2.

**Table 2:** Product description of “Shygys” patties.

1	Product name	Patties “Shygys”
2	Product description	A small (100 g) oval formed product produced from minced horse meat, horse fat, chicken meat (thighs), sprouted green buckwheat grain flour, spice, cabbage, onion, eggs
3	How it is to be used?	Fry in oil for 2–5 min each side
4	Packaging	Polyethylene bag, vacuum-packed
5	Shelf-life	Up to 3 months at −18 −20°C
6	Where it will be sold?	The product is sold at the retail store
7	Storage instructions	Keep at −18°C
8	Distribution conditions	In freezers at −18 −20°C
9	Consumers	Adults, children, elderly adults

### *Construction of the* Flow Diagram *(Chapter 8.5.1.5 of ISO 22000:2018)*

**Figure 3:**
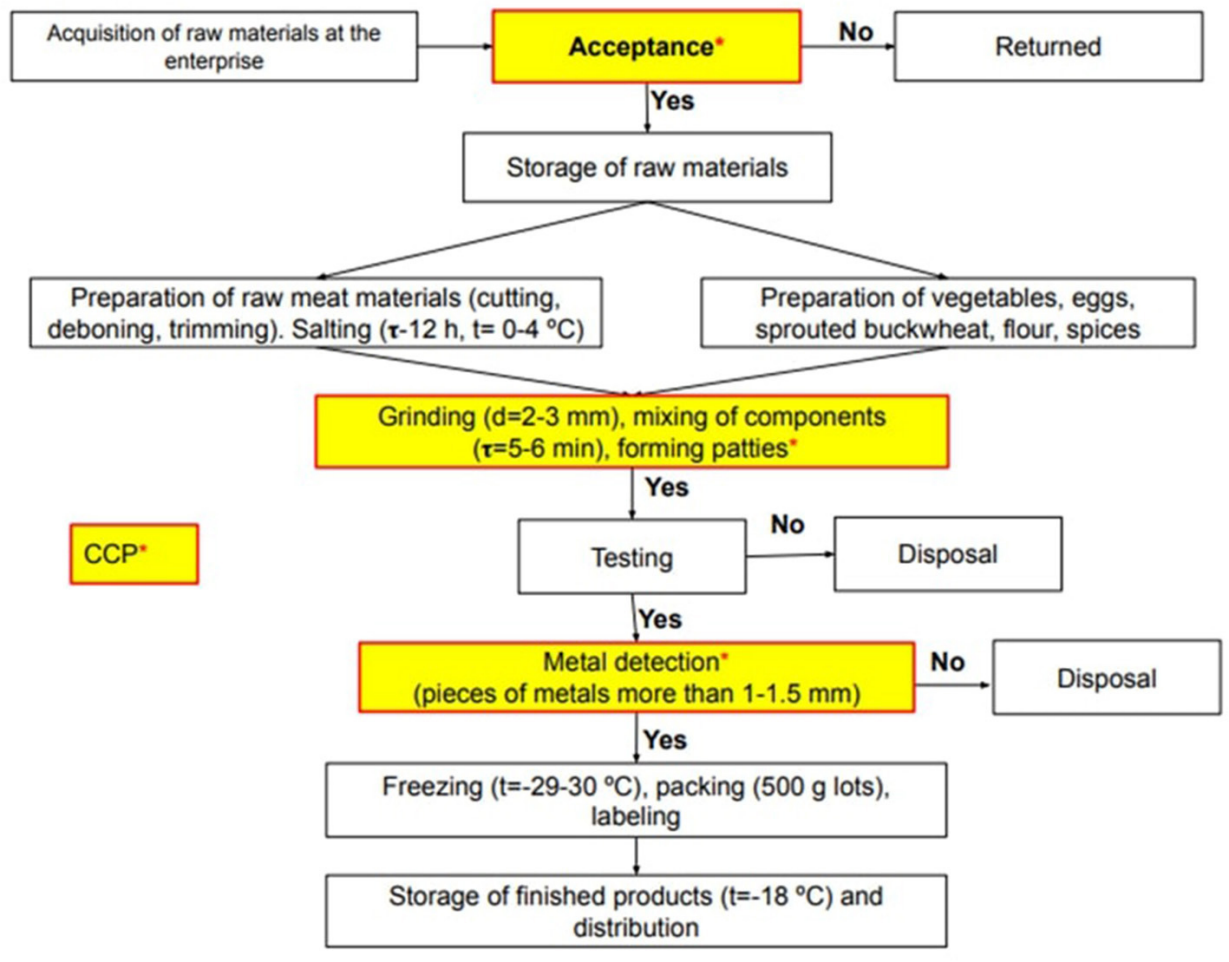
Flowchart of the production of the combined meat product “Shygys”.

The flow diagram mainly serves to help clear up the entire production process. It does not have to be complex or too simple and needs to be updated if necessary. The process flow diagram for the production line of the patties “Shygys” by “DARIA” SMPE is shown in Figure 3 and was inspected internally by the food safety team. In the flowchart, all the steps of preparation can be labeled as CCPs. Therefore as an initial step in developing a HACCP implementation strategy, it is necessary to implement PRPs that include good manufacturing practices (GMP) and fundamental hygiene conditions as cleaning procedures in the processing plant (32–34). Here, after testing the finished product for microbial and chemical toxins, if the quality of the final product does not follow the standard and cannot be reprocessed, the whole batch must be discarded once a specialist has reported and has recorded the information, and the corrective measures must be applied and the form filled out. Hence, each step of the process demonstrated in the flowchart should be recognized by the types of hazards, and the employees can make quick identification and be attentive to the possible occurrences of hazards. The flowchart must be reviewed if there were any abnormality occurrences, changes in the recipe, or customers' complaints according to Sections 6.2 and 6.3 of ISO 22000:2018.

#### Hazard Analysis and Determination of Acceptable Levels (Chapter 8.5.2 of ISO 22000:2018)

Hazard analysis was prepared by the HACCP team based on the HACCP checklist along with all the possible threats associated with untreated material, components, procedures, and a post-process operation were recognized and marked as biological (B), chemical (C), or physical (P). The hazards associated with various raw materials and production lines for patty manufacture and the determination of the control measures for each identified threat are shown in Table 3. Our study has considered the inevitability of determining the acceptance step as a CCP by using the risk analysis method in small- and medium-sized food enterprises aiming to follow the regulations and international standards. During the experiment, the HACCP system was strictly followed based on the identified critical control points (CCPs) presented on the process flow chart (Figure 3). Having well-answered the questions of the decision tree (Figure 2), the detailed procedure to minimize hazards was adopted to ensure the implementation of the HACCP (Table 3). ISO 22000 emphasizes risk assessment where a hazard assessment is recognized by assessing the risk associated with the severity (S) and possibility (P) of a threat (35–37). Each hazard is evaluated and gets a score between 1 and 3. The significance of hazard resulted from the hazard rating (HR) score from the multiplication of the severity by the probability is above three. Following the hazard identification, an HR is exhibited. For HR ≤ 2, the hazard is considered to be low or almost non-exists, and control measures are handled using PRPs, with no CP (control point) or CCP required (38). The risk assessments of the processing steps of horsemeat patties were determined after careful consideration of answers Q1 to Q4 of a decision tree that the steps during production of patties would either eliminate a hazard or lessen its risk to an acceptable level. Chemical hazards in the food industry are often related to heavy metals, pesticides, veterinary drugs, cleaning agents, allergens, and some food seasonings (39–41). Heavy metal impurities can contaminate meat products through the processing of raw materials, spices, packaging materials, and packaging methods and are a significant threat to food safety and human health due to the nature of the relatively low concentrations of toxins and/or their ability to accumulate in the body (25, 26, 42, 43). Physical hazards are considered to be equally crucial as chemical or biological hazards. They can occur through non-observance of PRPs or accidental contamination related to packaging or incorrect labeling.

**Table 3:** Hazards analysis and CCP determination chart of the patties production.

**Materials/ Process step**	**Category of Hazard**		**Is this hazard potentially significant?**	**Hazard assessment**			**Preventive measures/Control measures**	**CCP/ORPR/** **CP/NO**
				**S**	**P**	**HR**		
Acquisition of raw materials (meat, vegetables, eggs, spices, sprouted grain, flour)	B	*Salmonella, E coli, S.aureus, Listeria monocytogenes*, pathogens	**Yes** – possibility of a high microbial contamination in the final product due to a high initial presence in raw materials, which can lead to the cause of diseases	3	1	3	Application of strict standards; Requirement for providers to submit licenses for verification by sanitation management staffs; adequate receiving; temperature control, lab analysis sheet of rapid screening	**CCP1**
	P	foreign bodies due to improper wrapping	**No** – the presence of foreign bodies cannot cause injury to the consumer	2	1	2	Visual inspection, acceptance, and withdrawal by personnel; implementation of screening in consequent steps	CP
	C	antibiotics, grease, pesticide residue, allergens, toxins	**Yes** – Potential for chemical residues as a result of improper storage of raw materials or failure to remove them in sub-sequent steps.	3	1	3	Application of strict standards; Requirement for providers to submit licenses for verification by sanitation management staffs; adequate receiving; temperature control, lab analysis sheet of rapid screening	**CCP2**
Storage of raw materials	B	*Salmonella*, E coli, S.aureus, Listeria monocytogenes, pathogens	**Yes** – microbial contamination or growth due to improper storage	2	1	2	Correct setting of storage temperature, proper equipment setting; sanitation of all the transfer equipment; management according to Warehouse Control Standards and Operating Procedures	CP
	P	-	**No** significant hazards are presented	1	1	1		NO
	C	-	**No** significant hazards are presented	1	1	1		NO
Preparation of raw materials (grinding, chopping)	B	-	**No** significant hazards are presented	2	1	2	Proper personal hygiene and handling, clean, sanitize equipment. Management according to Production Processes Control Standards and Operating Processes	CP
	C	-	**No** significant hazards are presented	1	1	1		NO
	P	-	**No** significant hazards are presented	1	1	1		NO
Mixing, forming, freezing	B	*Salmonella, E coli, S.aureus, Listeria monocytogenes*, pathogens	**Yes-** possibility of microbial contamination due to improper processing; incorrect mixing	3	1	3		**CCP3**
	P	foreign bodies mixed in during operation processes; tools and parts of mechanical equipment mixed in due to breakage	**No**, no significant hazards are presented	2	1	2	Proof of reliability of mechanical equipment before and after use. Implementation of standard operating procedures for systematic maintenance and processes to avoid foreign bodies from being mixed in.	OPRP
	C	Cleaning agents	**No**, no significant hazards are presented	1	1	1	Application of strict standards; Requirement for vendors to submit licenses for verification by sanitation management staffs; adequate receiving; temperature control, lab analysis sheet of rapid screening	NO
Metal detection	B		**No**, no significant hazards are presented	1	1	1		NO
	P	foreign bodies mixed in during operation processes; tools and parts of mechanical equipment mixed in due to breakage	**Yes -** Unintended consumption of metal foreign bodies that can cause injury to the human body	3	1	3	All products have a metal detector test. The maintenance of the metal detector must be checked every quarter.	**CCP4**
	C		**No**, no significant hazards are presented	1	1	1		NO
Packing, labeling	B	Microbial growth due to inaccurate labeling, packaging, poor cleanliness and sanitation	**No**	2	1	2	Systematical inspection during labeling and packaging. Supervision according to Sanitation Management Standards and Operating Procedures, and Educational Training Standards and Operating Procedures.	CP
	P	Potential for misrepresentation, foreign material mixed because of improper packaging.	**No**	2	1	1	Extra control of information on label at each shift of product. Proper personal hygiene.	CP
	C	-	**No**	1	1	1		NO
Storage of finished product and distribution	B	-	**No**, no significant hazards are presented	1	1	1		NO
	P	-	**No**, no significant hazards are presented	1	1	1		NO
	C	-	**No**, no significant hazards are presented	1	1	1		NO

#### Selection and Classification of Control Measures (Chapter 8.5.2.4 of ISO 22000:2018)

As seen in Table 3, different biological, chemical, and physical hazards were identified in the process of receiving raw materials in the production of meat patties. The reliability of the ingredients at delivery is key to the safety of the final product. Hazards identified for unprocessed ingredients could be pathogenic bacteria (*Salmonella, E. coli*, and *S. aureus*), impurities (stones and plastic), and chemical hazards such as fertilizers, contaminations from antimicrobial solutions, and heavy metals. Control measures should be categorized following the PRPs or CCPs that are used with CCPs or OPRPs being subjected to improved control and classification based on observing occurrences. To guarantee the safety of untreated products, certified suppliers, trained personnel in good hygienic practices, and careful management of untreated products at receiving points are essential. If the raw material provider has not been certified by HACCP or ISO 22000 standards, chemical hazards may be designated as a CCP.

#### HACCP Plan (Critical Limits, Monitoring, Corrective Action, and Verification Procedure; Chapters 8.5.4, 8.7, and 8.9 of ISO 22000:2018)

It is recognized worldwide that the application of the HACCP system in food production has clear benefits in improving food safety and preventing foodborne infectious diseases. The work of HACCP focuses on specific measures to ensure food security that are achieved by the foundation and application of systems and the evolution of food security goals. During a hazard analysis control, the team took part in HACCP research and verified that all the hazard strategy identification activities were implemented according to PRPs, OPRPs, and HACCP plans. The HACCP plan model for this study is shown in Table 4.

**Table 4:** Hazard control plan (CCP plan) for production of patties “Shygys”.

**Processing/** **steps (CCPs)**	**Hazard description**	**Control limits**	**Monitoring**				**Corrective actions**	**Recording**	**Verification**		
			**Project**	**Method**	**Periodicity**	**Operator**			**Accountable person**	**Method**	**Periodicity**
CCP1, CCP2 – Acceptance	Biological, chemical	Coliform bacteria, TPC, *E.coli, Listeria*, coliform, Salmonella colonies count	TPC less than 2*10^2^, coliform >10^4^, Listeria not allowed in 25 g, Salmonella, not allowed in 25 g	Real-time rapid screening. Verification of the test report.	A sampling and analysis cycle is carried out for each batch. The rotation pattern is adjusted according to the intensity of the monitoring	Sanitation management personnel	Reject any dubious ingredients; corrections made based on the suppliers contract; sampling and testing.	Incoming materials control files; inconsistency correction record	Product study supervisor	Rapid screening record	Within 7 days
CCP3 - mixing, forming, freezing	Biological, physical, chemical	Microbial growth count. Foreign bodies and chemical contamination	TPC less than 2*10^2^, coliform >10^4^, Listeria not allowed in 25g, Salmonella, not allowed in 25 g	Real-time rapid screening. Checking of test report. Visual inspection, personnel hygiene	Each batch is exposed to one round of sampling and testing. Rotation plan is adjusted according to monitoring intensity	Sanitation management personnel	Reject or execute control receiving steps, control temperature of processing area	Quality record files	Product processing engineer	Microbial and visual test results, calibrating product testing and temperature	Every time
CCP 4 – metal detection	Physical, presence of metal substances	Metal detector sets: Cu ≤ 1.5 mm; Fe ≤ 1.0 mm; metals should not be spotted in final product	Metal foreign substances	All product are passed through the metal detector	Previous to startup of machine and at hourly intervals	Quality control engineer, onsite operation person	All pack materials and utensils are inspected for hygiene. If there any anomalies the onsite task person needs to respond and the quality control unit needs to find out the incident of metal particles and make sure that such an incident should not take place again. The production line must stop and maintenance personnel are warned. All products will be isolated. The operation person reexamine all the products. The anomalies record is updated.	Metal detector testing files, flaw tracking and progress, or inconsistency correction file	Quality control person, product research supervisor	Metal testing file is checked and the progress info is recorded in the production process quality control file verification	Every time

Physical contamination (foreign bodies) remains to be a threat to the food industry, even though there are many food safety management controls in place. As tools in food processing, the enterprise used a sifter (to separate and break up clumps in dry ingredients) and a metal detector (to detect foreign bodies mixed in during operation processes). In addition to quick screening methods, the enterprise strictly followed the Technical Regulations of the Customs Union “On Food Safety” (16) and regularly checked any changes in testing methods stated by the government, and after seeing monitored objects, periodic rotation procedures, and various observing schemes that factored in the non-conforming product contracts that were signed with producers and specified the terms for rejection and compensation. These procedures were performed to ensure the safety of the product for the consumer. The verification measures established by the food safety team were displayed to confirm that all components of the HACCP plan were successfully applied. It can be done by random sampling, in the form of audits, tests, and standardization of the apparatus used. Table 4 shows the critical limits, monitoring, corrective actions, and verification processes of each identified CCP during the manufacture of “Shygys” patties.

A study showed that HACCP principles not only guarantee the production of safe and high-quality products, record and increase the tracking of contamination sites, but thereby avert the further production of non-suitable foodstuffs, reduce the consumption of raw materials, financial and human resources, and increase the popularity of the enterprise in the food market (44, 45). The documented information may change over time if changes are made to a product, process, or any step in the process, or third-party audit results, or emergency cases. The enterprise must review the relevant documents and make the necessary changes to them, which will assist as a foundation for performance evaluation (Chapter 9) and improvement (Chapter 10).

#### Analytical Developments for the Implementation of HACCP and ISO 22000:2018 for the Patties “Shygys”

**Figure 4:**
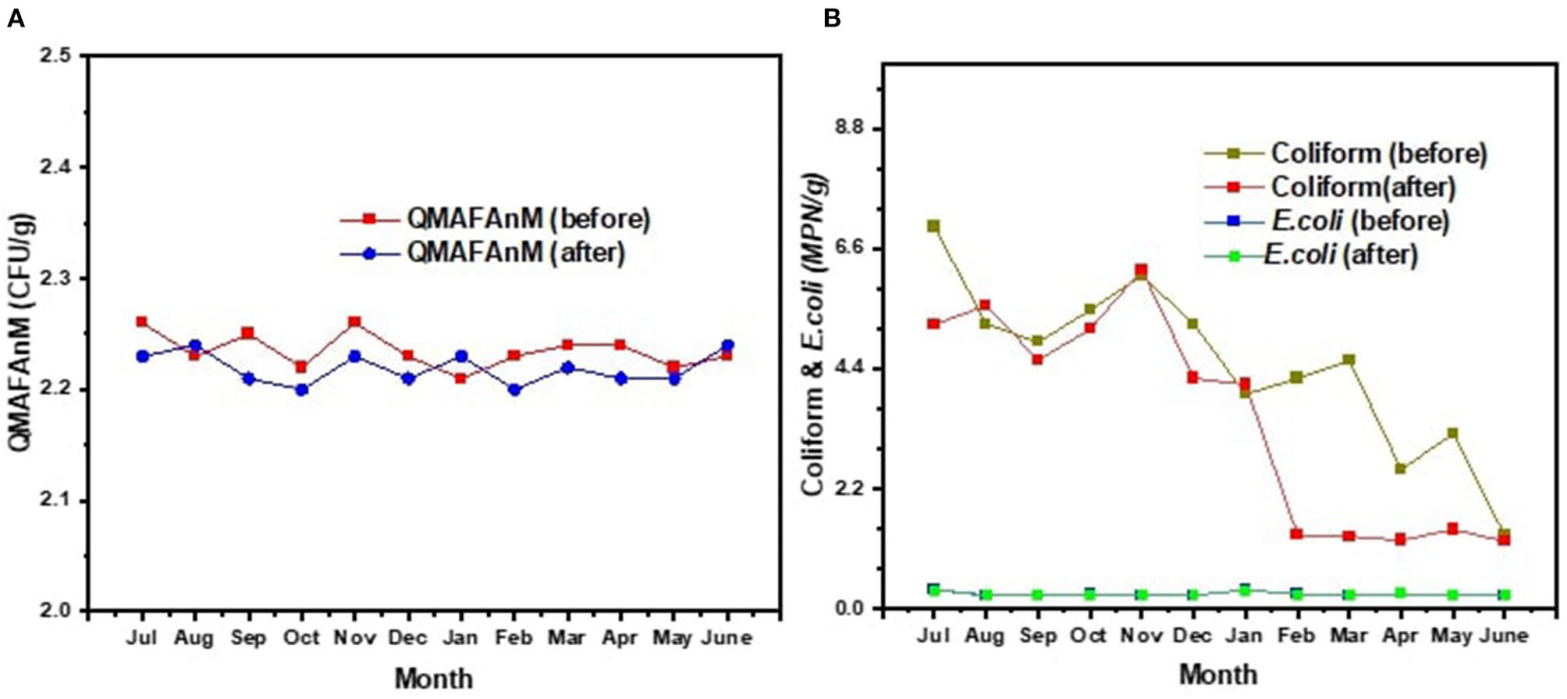
Microbial count analysis results **(A)** TPC; **(B)** Coliform & E.coli for semi-finished “Shygys” meat patties before and after the implementation of ISO 22000:2018.

Our study compares the data of the monthly average and total average for 1 year before and 1 year after the accomplishment of ISO 22000:2018. The analysis included the patties' TPC, coliform counts, *E. coli* counts, *Listeria monocytogenes* counts, pesticide residue level, the content of heavy metals, antibiotics level, the defect rate of the patties, and the number of inconsistencies needing correction. Figure 4 shows that there are no statistically compelling changes in the monthly microbial counts observed both before and after the implementation of ISO 22000:2018. In contrast, there was a statistically compelling difference in monthly coliform count (*p* < 0.05) that began to stabilize in February. It is probably that the “Acceptance” was determined as a CCP (biological hazard) and an agreement with suppliers, they applied strict controls over testing of pre-delivered raw materials; therefore, to minimize an item from being returned after failing a factory rapid tests inspection that was conducted after raw materials were delivered to the enterprise. A decrease in the microbial count after the implementation of HACCP and ISO 22000:2018 was reported by Arvanitoyannis et al. (36) and Jeffer et al. (38). Moreover, Jeffer et al. (38) noted that HACCP-based training and monitoring systems are needed in the whole supply chain to benefit public health and boost competitiveness.

**Figure 5:**
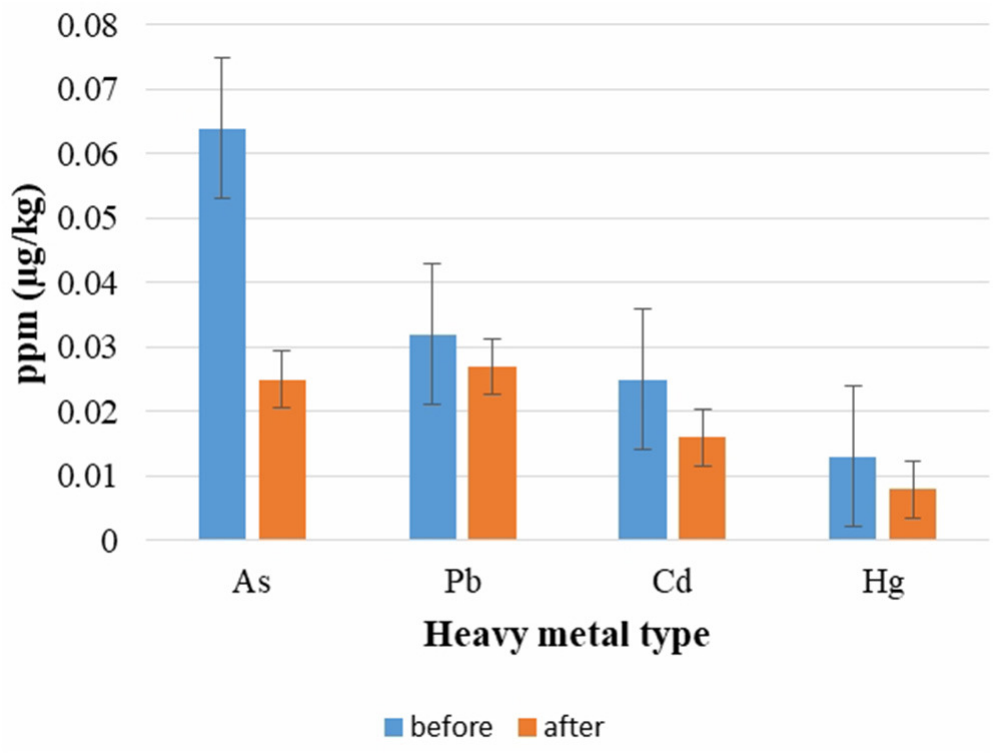
Testing results of heavy metals before and after implementation of ISO 22000:2018. *Considerably diverse between two groups of before and after implementation (*p* < 0.05).

**Figure 6:**
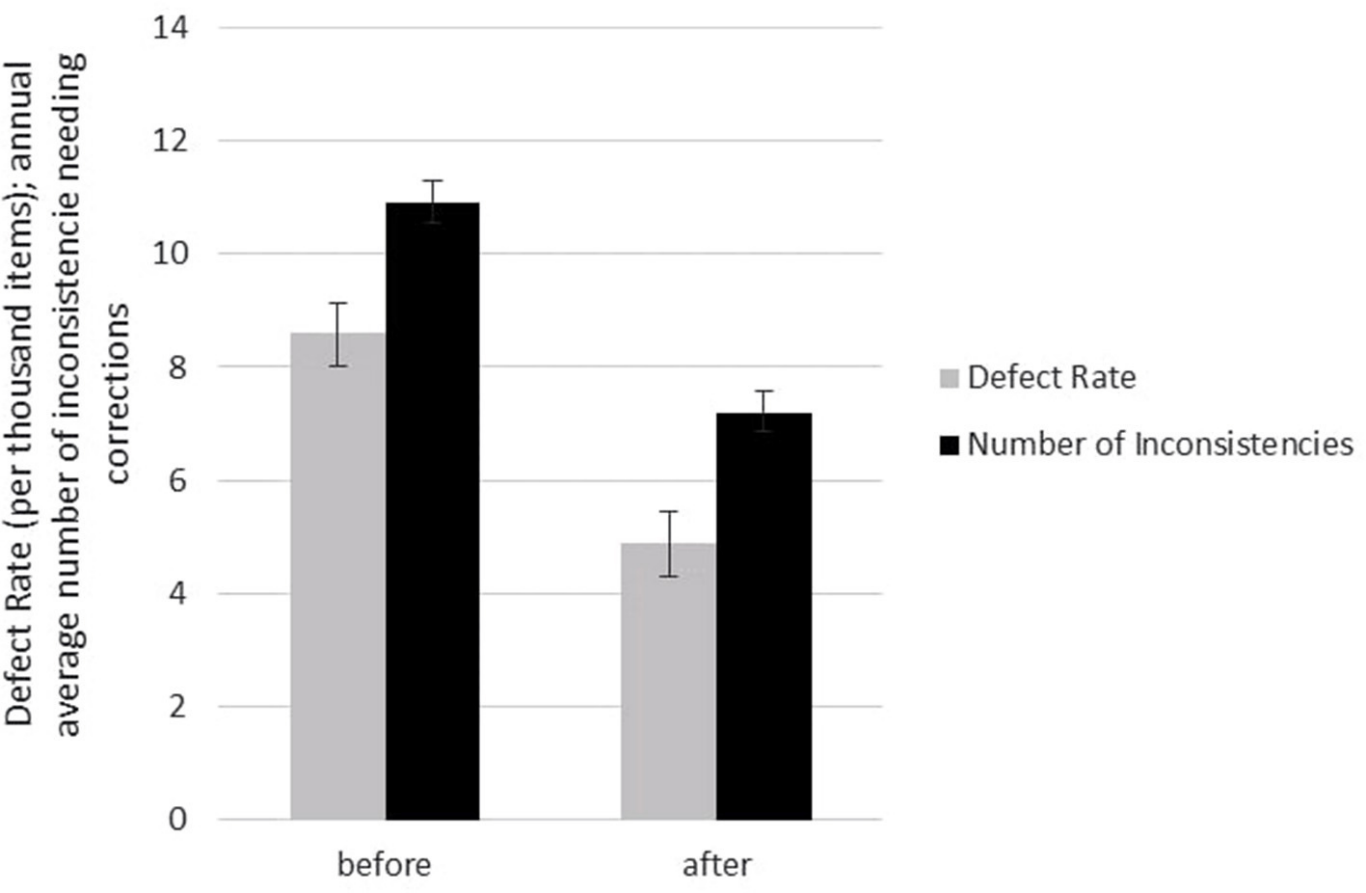
The changes in the product defect values and the amount of process flow inconsistencies needing improvement (before and after implementation of ISO 22000:2018).

The raw ingredients of the “Shygys” patties were tested negative for the presence of antibiotics and pesticide residues before and after the implementation of ISO 22000:2018; therefore, no data were shown in this work. The heavy metal test results are presented in Figure 5 before and after the implementation of ISO 22000:2018. The results described in this work are average examination results from samples obtained in July and October 2018 (before the implementation of ISO 22000:2018) and in July and October 2019 (after the implementation of ISO 22000:2018). It was ascertained that the content of heavy metals was at the maximum allowable concentration (MAC) levels according to Kazakhstan regulatory requirements before and after the application of ISO 22000:2018. It is important to note that a statistically substantial drop was discovered in terms of arsenic, cadmium, and mercury presence levels (*p* < 0.05); while there are not many differences observed in terms of lead content level. We can assume that the meat suppliers involved might have been motivated to meet the requirements of the assortment of meat after the description of “Acceptance – chemical hazard” as a CCP. Before implementing the ISO 22000 standards and HACCP principles, all the suppliers were informed about providing the relevant documents and notified that incoming raw materials would be tested quarterly for heavy metal content as part of a rotation plan for monitoring raw materials. This study has established that the implementation of ISO 22000 standards and HACCP principles helped to improve the concern of supplying satisfactory-level products to the enterprise. Cesium-137 was recorded as 5.4 ± 2.9627 Bq/kg in horsemeat and 6.7 ± 2.7045 in poultry. The activity of cesium-137 did not exceed the MAC. The test of heavy metal levels in the final product (data is not shown) – patty “Shygys” – showed a low detection level of the toxic elements both before and after implementation of ISO 22000:2018. Irrespective of the outcomes described above, the likelihood that meat and meat products exceed the standards for these indicators (MACs) cannot be ignored. Since environmental pollution due to these heavy metals increases every year, and the East Kazakhstan region of the Republic of Kazakhstan was the nuclear testing region for around 42 years, this could lead to the presence of these contaminants in the meat industry eventually (46). The analysis of toxic substances in the meat and meat products revealed that the average concentration of heavy metals did not exceed the respective MACs. The changes in the product defect values and the amount of process flow inconsistencies needing improvement are displayed in Figure 6. The defect values of “Shygys” patties are decreased, even though they were not statistically considerable. On the contrary, the number of process flow inconsistencies needing improvement during the production process statistically dropped (*p* < 0.05). These outcomes show that the implementation allowed us to monitor and influence suppliers to provide raw materials of high quality, decrease the amount of process flow inconsistencies needing improvement, and, with all that, increase the security and safety of the product's quality.

These results specify that acceptance of raw materials as a CCP labeled as a biological and chemical hazard resulted in unity with governing requirements and strengthened control over the raw material quality and showed that the implementation of food safety management systems is mandatory (47).

During the implementation of the system, we realized that it consumes extraordinary time to establish and implement HACCP and ISO 22000:2018 standards. So, one of our current study difficulties was research timing limitation: the research was conducted from 2018 to 2019; extending the time for research and analysis would reflect the benefits of FSMS implementation. However, the most prevalent benefits of the implementation of ISO 22000:2018 and HACCP were guaranteeing the assurance of consumers, improving company image, minimizing the possibilities of product contamination, safety hazards, and non-compliance with statutory requirements, followed by reaching market diversity through a cautious hazard control plan (identifications of CCPs) for the production process of patties. The manufacturer needs to focus on the process to ensure that the raw materials are consistently handled to produce patties with acceptable sensory-textural properties. The course of exertion of HACCP principles and ISO 22000:2018 could be arduous but achievable to be adopted in small industries with significant outcomes. Nonetheless, the results were shown to be statistically significant and, therefore, highly reliable for those food companies intending to learn more about implementing the ISO 22000 model of food safety management system.

Future study is needed to evaluate the impact of investment in quality to increase the overall profits.

## Conclusion

In this study, a newly developed product was applied in a small-scale enterprise to meet the requirements of ISO and HACCP standards. The receiving of raw materials, heavy metals, and radionuclide detection components of the production process for “Shygys” patties were considered as CCPs, and analysis of various ingredients showed that the application of ISO 22000:2018 and the HACCP principles can improve the quality of the finished product. The study only surveyed the implementation and processing settings of ISO 22000:2018. This model can be used as a risk calculation plan for foreseeing chemical, physical, and biological hazards of raw materials and as a basis for further examinations of food safety management systems. Through the results obtained in this study, the researcher could perceive a significant improvement after the implementation of ISO 22000:2018 and the HACCP plan regarding the processing of patties in a small-scale enterprise. It could be valuable in different food productions, specifically for businesses with no testing facilities. During 1 year, the enterprise noticed the defect values of “Shygys” patties had decreased, even though it was not statistically significant, which showed that the strict implementation of standards allows the company to ensure the safety of incoming materials and, in the end, to get a safe and finest product. The continuous control of the rotation plan needs systematic outsourced testing and therefore increases testing costs. The only following part of this plan helped the enterprise minimize the occurrence of contamination of incoming materials and, therefore, increase the quality of the final product and, with that, meet customer's demand. In addition, the enterprise's determination to improve the safety of its product “Shygys” patties had practical implications and helped to increase its discernibility and sales and create a food safety management system that meets ISO 22000 and HACCP criteria. The implementation of the international standards will benefit any enterprise with access to new markets, both internal and external; increasing productivity and competitive advantage; better quality products according to customer preferences; improving reporting and communications; and moreover, bringing a new product into the market means delivering new value to the customer.

## Data Availability Statement

The original contributions presented in the study are included in the article/supplementary material, further inquiries can be directed to the corresponding author/s.

## Ethics Statement

The studies involving human participants were reviewed and approved by the Ethics Committee of Shakarim State University, Semey, Kazakhstan (protocol #54). The participants provided their written agreement to participate in this study. All suppliers have been informed before study and enrolled in the project.

## Author Contributions

Conceptualization: ZhA and AN. Methodology: ZhA, MR, and AN. Software: SK and DS. Validation: ZhA, MR, and AN. Formal analysis: ZhA, RA, SK, GK, and ST. Writing—original draft preparation and review and editing: ZhA, AN, and ZA. Supervision: MR. All authors have read and agreed to the published version of the manuscript.

## Conflict of Interest

The authors declare that the research was conducted in the absence of any commercial or financial relationships that could be construed as a potential conflict of interest.

## Publisher's Note

All claims expressed in this article are solely those of the authors and do not necessarily represent those of their affiliated organizations, or those of the publisher, the editors and the reviewers. Any product that may be evaluated in this article, or claim that may be made by its manufacturer, is not guaranteed or endorsed by the publisher.
